# Supplementary material for: Efficient generation of human primordial germ cell-like cells from pluripotent stem cells in a methylcellulose-based 3D system at large scale
Source: PeerJ. 2019 Jan 9;6:e6143. doi: 10.7717/peerj.6143 (PMC6330037; doi:10.7717/peerj.6143)
Supplement: Supplemental Information 1 [file peerj-07-6143-s001.docx]

**Table S1.** Q-PCR Primers

| Gene | Forward primer | Reverse primer |
| --- | --- | --- |
| BLIMP1 | AAACCAAAGCATCACGTTGACA | GGATGGATGGTGAGAGAAGCAA |
| TFAP2C | ATTAAGAGGATGCTGGGCTCTG | CACTGTACTGCACACTCACCTT |
| NANOS3 | TGGCAAGGGAAGAGCTGAAATC | TTATTGAGGGCTGACTGGATGC |
| DAZL | TGGCCCTTCTTTCAGTGACTTC | GACCCTAGGGGGCACTAGTAA |
| DPPA3 | AAGCCCAAAGTCAGTGAGATGA | GCTATAGCCCAACTACCTAATGC |
| DDX4 | TTCTTCACAAGCTCCCAATCCA | TTCTTCTCTGCATCAAAACCACA |
| PRDM14 | TATCATACTGTGCACTTGGCAGAA | AGCAACTGGGACTACAGGTTTGT |
| KLF2 | ACTAGAGGATCGAGGCTTGTGA | TGCCCACCTGTCTCTCTATGTA |
| KLF4 | AGCCTAAATGATGGTGCTTGGT | CCTTGTCAAAGTATGCAGCAGT |
| ESRRB | TAAAATGGCAGTTCCCCATTGC | CCAGATACATGGGACCAGGATG |
| POU5F1 | CTGTCTCCGTCACCACTCTG | AAACCCTGGCACAAACTCCA |
| NANOG | AGAGGTCTCGTATTTGCTGCAT | AAACACTCGGTGAAATCAGGGT |
| SOX2 | TGAATCAGTCTGCCGAGAATCC | TCTCAAACTGTGCATAATGGAGT |
| SOX17 | TTCGTGTGCAAGCCTGAGAT | TAATATACCGCGGAGCTGGC |
| GATA4 | CCTCTTTCTCAGCAGAGCTGTA | CTCTGCTACAGCCAGTAGGATT |
| GATA6 | ACAGGGCGATTTCCTTTCAGTT | CTTCTGTTGGGGGTAACGTCTG |
| NODAL | CATTGCCTCAGGCTGGGTTG | GTACAGCTCATTAGCAGAGAACCA |
| T | AGCCAAAGACAATCAGCAGAAA | CACAAAAGGAGGGGCTTCACTA |
| DNMT3A | TGGGATTCATCCAGACTCATGC | AAAGTGAGAAACTGGGCCTGAA |
| DNMT3B | TAACTGGAGCCACGACGTAAC | GCATCCGTCATCTTTCAGCCTA |
| ARBP | GAAACTCTGCATTCTCGCTTCC | ACTCGTTTGTACCCGTTGATGA |
