# Supplementary figures and images for: Efficient generation of human primordial germ cell-like cells from pluripotent stem cells in a methylcellulose-based 3D system at large scale
Source: PeerJ. 2019 Jan 9;6:e6143. doi: 10.7717/peerj.6143 (PMC6330037; doi:10.7717/peerj.6143)

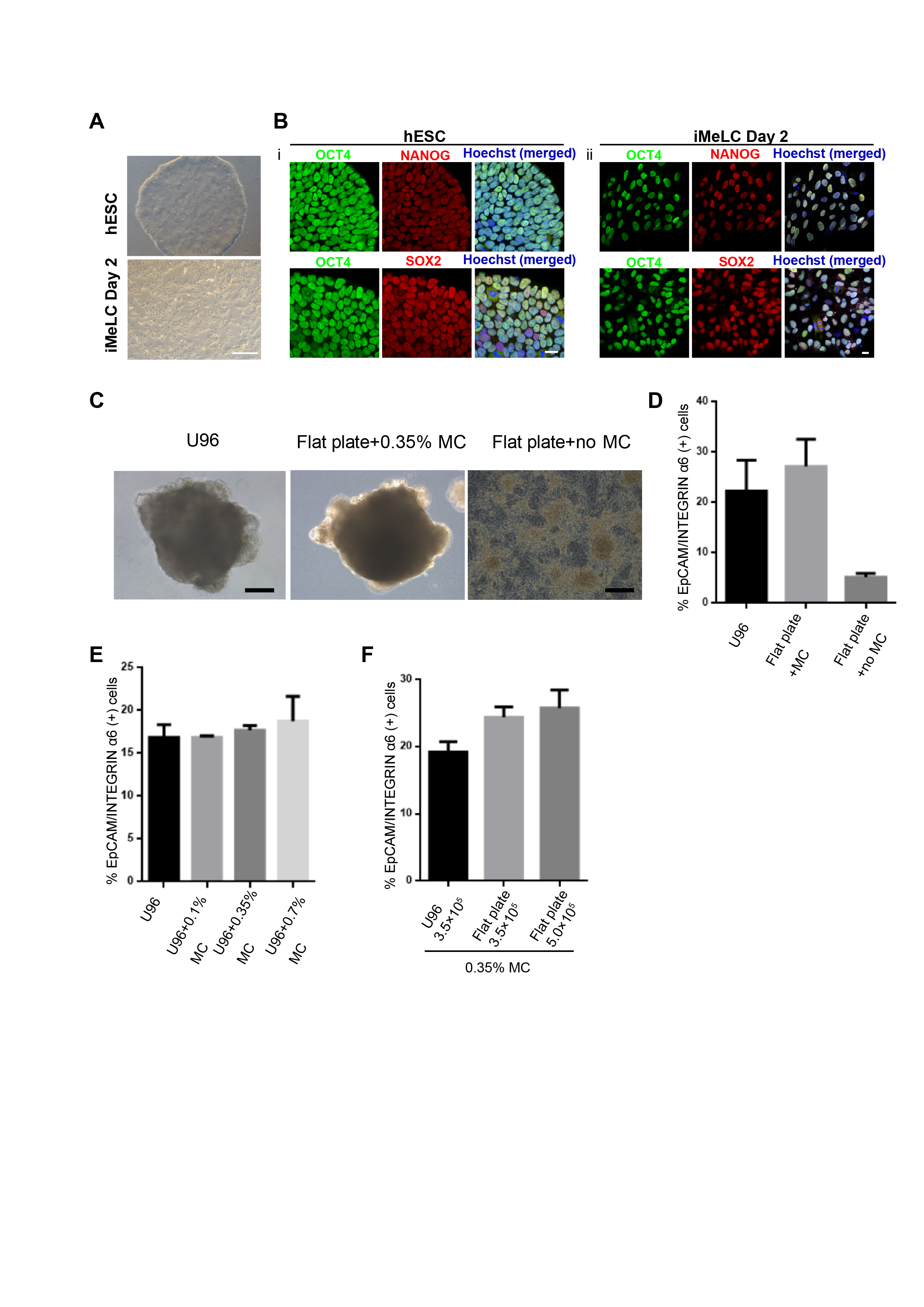

Supplement: Supplemental Information 2 — (A) Phase-contrast image s of Fy-hES-3 cells (top) and iMeLCs (bottom). Scale bar, 500 μm. (B) Immunostaining of OCT4, SOX2 and NANOG in Fy-hES-3 cells (top) and iMeLCs (bottom). The nuclei were stained with Hoechst. Scale bar in the left, 50 μm , Scale bar in the right, 20 μm. (C) The typical morphology of EBs generated via U96 method, flat plate with 0.35% MC method and flat plate without 0.35% MC, respectively. Scale Bars, 200 μm. (D) The proportion of EpCAM-/INTEGRINα6-high cells of day 4 EBs via U96 method, flat plate with 0.35% MC method and flat plate without MC. Two replicates were performed. (E) The proportion of EpCAM-/INTEGRINα6-high cells from Fy-hES-3 at day 4 via U96 method and the addition of MC based on U96 method. Two replicates were performed. (F) The proportion of EpCAM-/INTEGRINα6-high cells from Fy-hES-3 at day 4 via U96 + 0.35% MC method and 0.35% MC methods (different seeding numbers). Two replicates were performed. [file peerj-07-6143-s002.png]

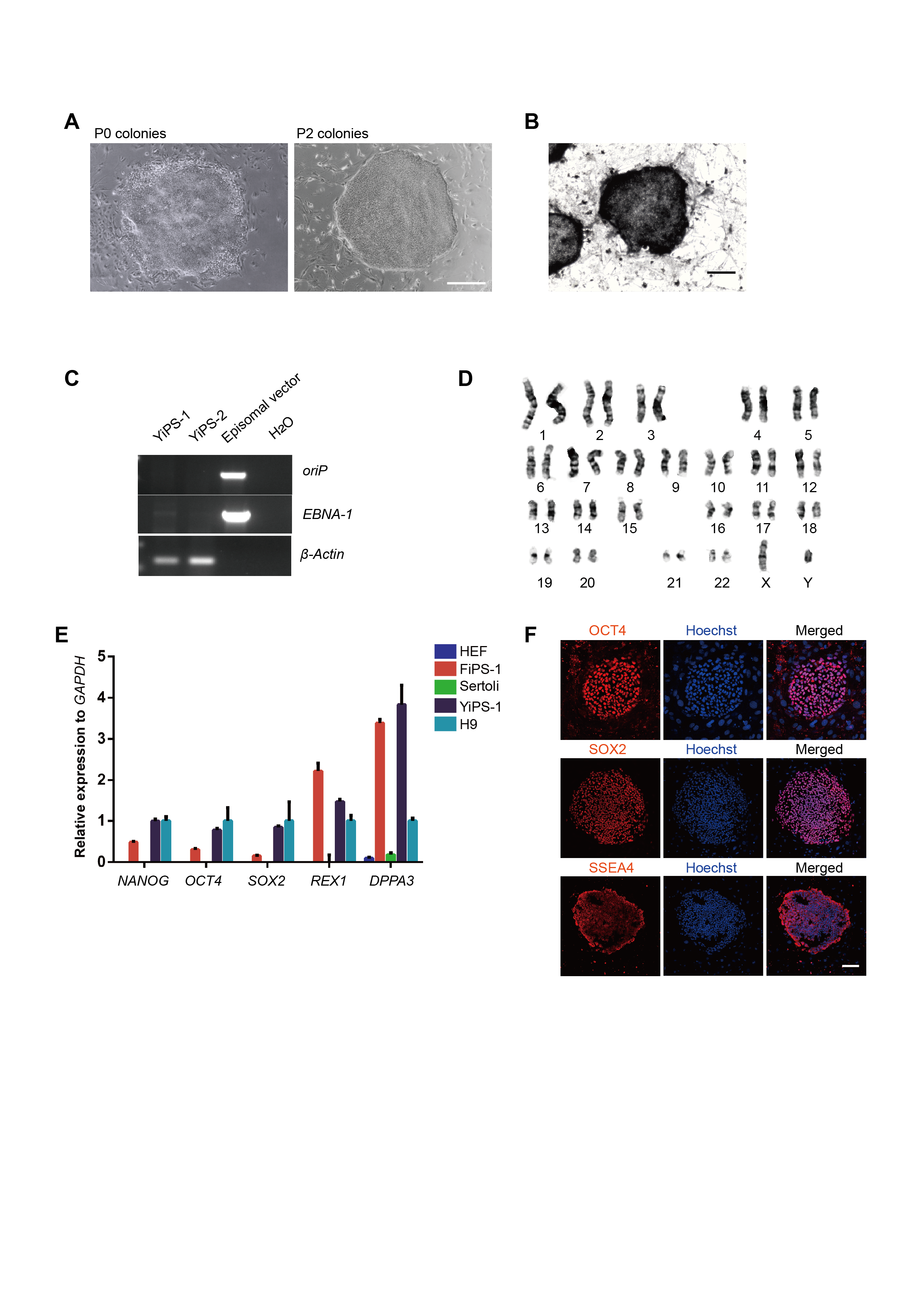

Supplement: Supplemental Information 3 — (A) The P0 (left) and P2 (right) colonies of YiPS cells showed typical hES-like morphology. Scale bar, 500 μm. (B) AP staining of YiPS cells. Scale bars, 500 μm. (C) Detection of the expression of oriP and EBNA-1 in two YiPSCs lines. (D) Karyotype analysis of YiPS cells. (E) Quantitative analyses of pluripotency-related markers. HEF, Human Embryonic Fibroblast. H9, H9 hESC. (F) Immunostaining of OCT4, SOX2 and SSEA4 in YiPS cells. The nuclei were stained by DAPI. Scale bar, 100 μm. [file peerj-07-6143-s003.png]

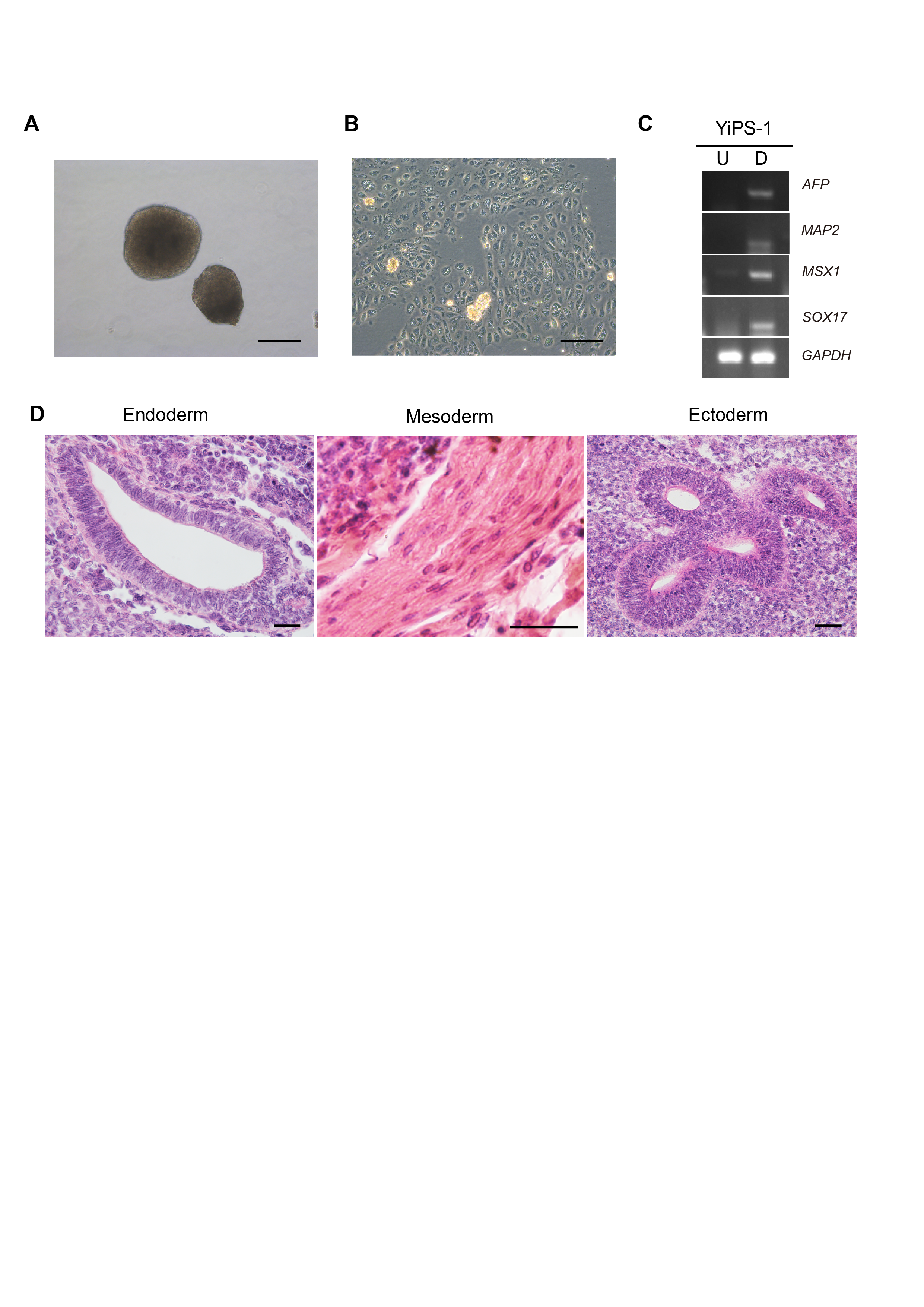

Supplement: Supplemental Information 4 — (A) EBs at day 8 derived from YiPS-1. Scale bar, 200 μm. (B) The morpholgy of differentiated cells from YiPS-1 via EB-based differentiation strategy at day 16. Scale bar, 200 μm. (C) The expression of marker genes of three embryonic layers in the differentiated cells derived from YiPS-1. U, undifferentiated cells. D, differentiated cells. (D) HE staining of the teratoma sections derived from YiPS-1. The teratoma tissues contained gut-like epithelium (endoderm, left), striated muscle (mesoderm, middle) and rosettes of neural epithelium (ectoderm, right). Scale bars, 50 μm. [file peerj-07-6143-s004.png]

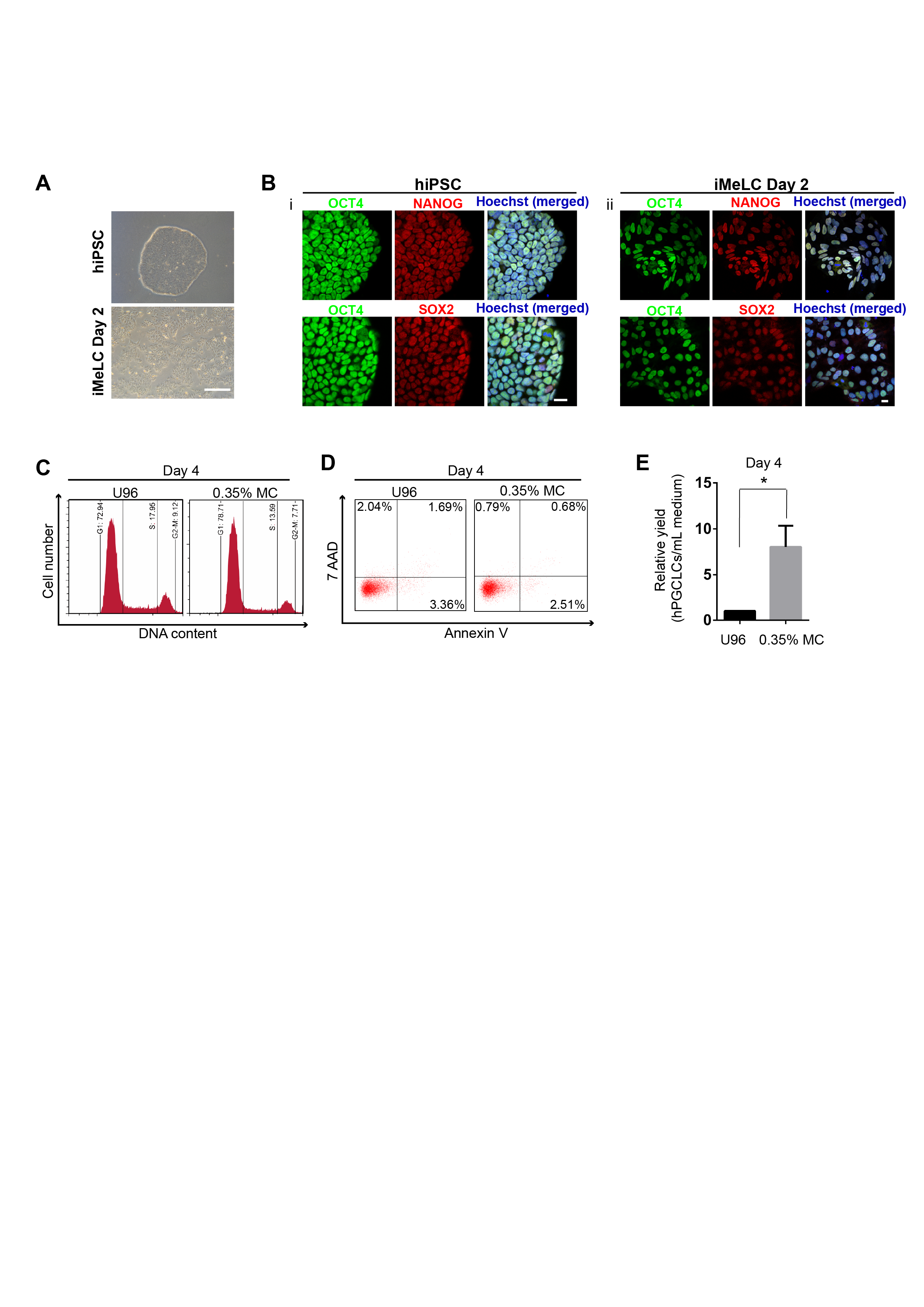

Supplement: Supplemental Information 5 — (A) Phase-contrast image of YiPS-1(top) and YiPS-1-derived iMeLCs (bottom). Scale bar, 500 μm. (B) Immunostaining for OCT4, SOX2 and NANOG of YiPS-1 (top) and YiPS-1-iMeLCs (bottom). The nuclei were stained with Hoechst. Scale bars, 20 μm. (C) FACS analysis of cell cycle states of day 4 EBs via U96 method and 0.35% MC method. (D) FACS analysis of apoptosis from day 4 EBs via U96 method and 0.35% MC method. (E) The relative efficiency of the yielded hPGCLCs from per ml hPGCLC medium via U96 method and 0.35% MC method. The number of hPGCLCs from U96 plate was set to 1 for reference. *P < 0.05. [file peerj-07-6143-s005.png]
